# A qualitative exploration of the sociology of poststroke visual impairments and the associated health inequalities

**DOI:** 10.1002/brb3.1738

**Published:** 2020-06-26

**Authors:** Kerry Hanna, David Mercer, Fiona Rowe

**Affiliations:** ^1^ Department of Health Services Research University of Liverpool Liverpool UK

**Keywords:** health inequalities, qualitative, sociology, stroke, visual impairment

## Abstract

**Introduction:**

Inequalities have been found to exist within the visually impaired stroke population on an individual level, in relation to demographic differences, unequal access to vision services, transport, employment, and financial repercussions. The long‐term impact of living with poststroke visual impairments must be explored, in order to identify complications accessing NHS services and to inform possible changes to service planning and delivery in order to tackle such complications.

**Aim:**

To explore the extent of health inequalities within visually impaired stroke survivors in the northwest of England and discuss potential solutions to these.

**Methods:**

Focus groups and individual interviews (*n* = 13 stroke survivors and *n* = 1 spouse) were conducted between October 2016 and January 2017. Transcription and thematic analysis of the transcripts was undertaken, using line‐by‐line coding, underpinned with social constructionism.

**Results:**

The findings draw on lived experiences of stroke survivors across their journey from prestroke to life after stroke. The three overarching experiences of stroke and visual impairment that emerged in respondent accounts were constructed in terms of “loss,” concerning (a) the physical being, (b) the psychosocial being and, (c) the systematic organization of health care.

**Conclusion:**

The stroke survivors frequently reported a complete lack of visual care, with many recounting apathetic experiences, often resonating power imbalance in the healthcare system. Where suitable care is being offered after stroke, a desire for a personalized approach to rehabilitation, with adapted communication methods specific to individual needs, featured strongly in many of the respondent accounts. The findings emphasize a need to ensure vision rehabilitation is offered to all stroke survivors suffering from poststroke visual impairment, and to educate stroke clinicians and patients of the bigger picture of life after stroke, highlighting all forms of available support.

## Graphical abstract

The long‐term impact of living with poststroke visual impairments has been explored using focus groups and individual interviews, in order to identify health inequalities and potential means of addressing these. The findings emphasize a need to educate clinicians and patients of the bigger picture of life after stroke, highlighting all available support.

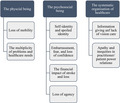

## 1 BACKGROUND

Earlier reports concluded that inequalities stem from lack of education, poor working conditions, poor transport links, unacceptable housing, children's start in life, unhealthy food and lifestyle, and the role of the NHS/public sector (Acheson, 1998; Black, 1980; Marmot, 2010). However, explanations for these social health inequalities have gradually moved away from a “neomaterialist theory” that links the impact of material possessions to public health through access to goods, services, and material risk factors (Skalická, van Lenthe, Bambra, Krokstad, & Mackenbach, 2009). Instead, subsequent researchers have postulated a “psychosocial perceptions” theory to explain social inequalities, whereby people of lower social standing feel inferior and subordinated, and the subsequent impact of these negative feelings on their health (Marmot & Wilkinson, 2001; Skalická et al., 2009). Furthermore, the “psychosocial pathway” links income inequality and population health through the biology of chronic stress (Marmot & Wilkinson, 2001). This theory states that living in unhealthy and impoverished environments increases stress levels markedly, which, in turn, increases unhealthy risk behaviors, such as smoking and drinking (Wilkinson, 2002). In stroke research, the role of lifestyle stresses has been significant in developing stroke, although less is known of the impact of stresses after stroke (Backé, Seidler, Latza, Rossnagel, & Schumann, 2012). Visual impairments suffered in nonstroke groups have resulted in emotional challenges through loss of ability to undertake tasks and hobbies (Teitelman & Copolillo, 2005), suggesting those suffering from poststroke visual impairments may suffer similar challenges.

The psychosocial impact of poor health and living with visible disabilities has been reported previously. The stigma of living with a “spoiled identity” (Goffman, 1968), such as the visible impairments of stroke, has been found to result in social isolation and even depression (Pallesen, 2014). Furthermore, inequalities may exist within the visually impaired stroke population on an individual level, in relation to demographic differences. A recent systematic review reporting health inequalities within the visually impaired stroke population concluded a lack of specific evidence within this area (Hanna & Rowe, 2017). However, unequal access to vision services after stroke based on area of residence was identified and called for national improvement (Rowe, 2013), although qualitative exploration is required to explore the views from this group specifically, in terms of health inequalities and psychosocial impact.

Furthermore, stroke and visually impaired groups individually presented with health inequalities (Hanna & Rowe, 2017), such as increased prevalence of stroke, poor recovery of impairments, and poor access to health services in subgroups such as older age, female gender, and lower socioeconomic status (Cumberland, Rahi, & UK Biobank Eye and Vision Consortium, 2016; Knight & Lindfield, 2015; Redfern, McKevitt, Rudd, & Wolfe, 2002). Therefore, it is possible that the combined group are facing issues that have not been fully explored.

Within visually impaired stroke groups, little is known regarding the psychosocial impact of vision impairments, which are contrastingly invisible to the public, although work has discussed “hidden disabilities” in other forms that have presented with separate difficulties, including the inability to interact successfully with others without continuously explaining oneself (Carlsson, Möller, & Blomstrand, 2009). With new‐onset visual impairments occurring in 60% of stroke survivors (Rowe et al., 2019), it is likely that such adversities are far‐reaching and underreported.

Therefore, the collective physical and psychosocial impact of suffering vision impairments after stroke, living with both a spoiled identity and hidden disabilities, is currently unknown. The aim of this qualitative research, therefore, was to explore the lived experiences of visually impaired stroke survivors and gain an insight into the impact on their everyday lives, with an overarching focus on their health and well‐being. Such information can inform healthcare providers of the most effective way to care for these patients by addressing all potential poststroke adversities.

## 2 METHODS

This research presents the findings from individual interviews and focus groups with visually impaired stroke survivors. This research adopts a social constructionist ideology, exploring the lived experiences of stroke survivors through narratives and language, and attempts to neutralize power imbalance by giving them a voice in a way that quantitative research cannot. Green and Thorogood (2018) reported a longstanding tradition in the social sciences that considers a positivist view to be unachievable and inappropriate when researching human behavior compared to natural science, as humans make sense of their place in the world, have views about the researchers studying them, and behave in unpredictable ways. The authors further deduced a preference for the tradition of constructionism in qualitative health and social sciences research, although they acknowledge that there is no definitive approach to research (Green & Thorogood, 2018). A social constructionist approach encourages the researcher to accept that reality is an outcome of the human processes, thus including open questioning that may not necessarily be considered core to the research, but generates relevant findings that explain the lived experiences of the participants (Green & Thorogood, 2018). This school of thought informs the current research that the knowledge extracted from the qualitative research is actively constructed by the interviewed stroke survivors, rather than being passively received by them.

Considering this definition, this research focuses on understanding the lived experiences from the visually impaired stroke survivors from their points of view. The work undertaken has been developed through knowledge constructed during the research and interview process, while drawing on social theory and a previous knowledge of health inequalities.

Ethical approval to conduct the research was sought through the University of Liverpool's Research Ethics Committee. Informed written consent was obtained from all participants. One author (KH) recruited the participants and conducted the focus groups and individual interviews. As experience in qualitative interviewing can impact on whether rich data will be obtained, the author (KH) underwent additional training and was, further, closely supported on this work by the other highly experienced authors. Two authors (KH and DM) conducted the analysis, and all authors contributed to the writing of this article.

### 2.1 Sample selection (inclusion and exclusion criteria)

Any stroke survivor with a visual impairment as a direct cause of stroke was invited to take part in a focus group or interview. Potential participants disclosed their visual impairments to the researcher (KH) on expression of interest. On discussion, visual impairments were classified and confirmed to align with stroke onset. Visual impairments included reduced visual acuity, visual field loss, ocular motility disorders, and/or visual perceptual disorders. All participants were adults aged ≥ 18 years old, as younger stroke survivors would likely possess differing experiences due to differences in stroke causation (Ganesan et al., 2000; Williams, Garg, Cohen, Fleck, & Biller, 1997). All ethnicities, ages, and genders were included, and participants must have been admitted to a stroke unit in the northwest of England (NWE). Those with severe dysphasia/aphasia, but who met the inclusion criteria to participate, were still invited to take part provided means of translation/communication could be used.

### 2.2 Recruitment and participants

Recruitment was made through various stroke charities and group meetings. Participants were invited to take part in focus groups; however, it became apparent during recruitment that the stroke disabilities prevented many participants from attending a focus group. This inability to partake in a focus group highlighted potential inequalities and the importance of including these participants in the research. Therefore, to limit the risk of total exclusion from the research, individual interviews were offered to these stroke survivors in a convenient location for them. Previous research has identified similar difficulties in recruiting participants to focus groups resulting in a “substitution” of individual interviews (Happell, 2007). It is suggested that choosing a time and location that best suits the participants can overcome such difficulties. In the case of this research, despite originally agreeing on a suitable time and location, the individual and physical needs of the participants were variable and unpredictable. Therefore, a flexible approach had to be used to accommodate their requirements to ensure inclusion in the research, by offering alternative interviews (Knodel, 1993).

The recommended numbers for focus groups vary from as little as four participants (Kitzinger, 1995) to as many as 15 (Goss & Leinbach, 1996). It was decided that an average of four to six participants would be included in each group, in order to accommodate the expressive dysphasia disability of many of the participants. The aim of interviewing a smaller number of people per group was to allow each stroke survivor ample time in discussions while keeping to the recommended time frame: typically no more than two hours of duration (Liamputtong, 2011).

The interviewer had no pre‐existing relationship with any recruited participant.

### 2.3 Conducting the focus groups and interviews

A predesigned topic guide (Krueger, 1994) was used to support semi‐structured interview questions around the participants’ type of stroke/visual impairments, their experiences of accessing health services, and their accounts of life after stroke.

#### 2.3.1 Environment and settings

It is important to note the variable settings in which the interviews and focus groups took place (Gagnon, Jacob, & McCabe, 2015; Herzog, 2005). It was essential that the participants felt comfortable to share their thoughts, especially around sensitive or personal topics (Happell, 2007), such as the individual nature of their stroke condition.

For those recruited through stroke charities, the participants expressed a desire to remain at the community centers after their weekly meeting had finished, which would prevent an additional trip to an unfamiliar location. Thus, two focus groups and one of the interviews took place in a community center.

The first focus group was held in a comfortable, well‐lit room, most often used for arts and crafts activities. The second focus group was held in a boardroom, as the first room was unavailable. The three male participants spread themselves out around a large meeting table, despite being asked to sit close together at one end. Reasons for this may include their acquired physical impairments that required aids such as wheelchairs and walking sticks that took up more space around the table. Another possible reason for their dispersed seating was the fact that the three participants were not “friends” outside of the stroke meeting (unlike many of the female participants of the first focus group). It was clear before the second focus group began that the men did not feel as comfortable with one another, and the interviewer was aware that their engagement within the focus group could be affected. The interviewer therefore had to employ methods to encourage conversation and attempt to neutralize differences caused by the variability in the room environments (Kitzinger, 1995). Furniture was repositioned to form a circle for group discussion, and audio recorders were placed on either side of the table to ensure that their voices were heard and captured.

One interview was conducted in the community center, and the rest were conducted in the patients’ homes. The variable settings for the focus groups and interviews each posed individual implications to conducting research. However, these were acknowledged and addressed, where possible, by the interviewer to ensure interviews and focus groups were conducted as consistently as possible. Community organizations, such as the stroke charity organization centers, act as a middle ground between healthcare settings and home settings (Gagnon et al., 2015). It allows the participants to view the interviewer, not as a clinician, but as an interested researcher, which might encourage confidence in discussing personal issues (Gagnon et al., 2015). Privacy has been suggested as a possible complication when conducting interviews in community settings, although this issue did present itself during the research study. The interviews conducted at the participants’ homes were deemed most convenient for the participant; however, this was not always the case for the interviewer. Additional travel was required to complete the interviews across the NWE, and a lone‐worker policy had to be followed to ensure interviewer safety. However, providing patients with a choice on where they would like to be interviewed provides an “equal relationship” and a safe space to share personal experiences (Gagnon et al., 2015).

#### 2.3.2 Transcription and analysis

The audio recordings were transcribed into verbatim scripts by both KH and a paid typist from the university with extensive transcribing experience. Completed transcripts were reread while listening to the audio recording twice to ensure thorough transcription had been achieved before undertaking analysis.

Line‐by‐line, manual coding was employed individually by two authors to evaluate the transcripts and extract codes. Transcript‐based analysis was followed, such that transcripts were read and coded one at a time to identify emerging themes and categorize codes (Braun & Clarke, 2006; Krueger, 1994). Later, both authors met to discuss individual analyses and converging themes were established using the NVivo 10 qualitative software package (QSR International Ply, 2012), and agreed by all authors.

## 3 RESULTS

Overall, two focus groups and five individual interviews (13 stroke survivors and 1 spouse) were conducted in three areas in the NWE between October 2016 and January 2017. The demographics of the recruited participants are shown in Table 1. The findings presented draw on lived experiences of stroke survivors across their journey from prestroke to life after stroke. Table 1 shows the poststroke visual impairments suffered by each of the participants. The first focus group consisted of five participants, all female. No one with complete aphasia was recruited; however, two‐stroke survivors with dysphasia participated with translational support, one with their spouse and the other with their friend (another stroke survivor who was independently recruited to the study): See Table 1.

**Table 1:** Demographics of research participants

Anonymous identifier	Interview style	Gender	Ethnicity	Age (at time of interview)	Year of stroke	Vision defect	Other stroke impairments
Respondent 1	FG1	F	Black British	60	2013 and 2015	OM	Gait, memory loss
Respondent 2	FG1	F	WB	46	2010	OM, VF	Memory loss
Respondent 3	FG1	F	WB	52	2012	VF	Dysphasia^a^
Respondent 4	FG1	F	WB	39	2014	VF	Dysphasia
Respondent 5	FG1	F	WB	65	1979	VF	Dysphasia
Respondent 6	FG2	M	WB	53	2012 and 2014	VF	Gait
Respondent 7	FG2	M	WB	53	2006	OM	Memory loss
Respondent 8	FG2	M	WB	54	2010	OM, VF	Dysphasia, reduced cognition
Respondent 9	Interview	M	WB	57	2006 and 2010	VF, VA	Reduced cognition, hemiparesis, gait, memory loss
Respondent 10	Interview	M	WB	65	2005	VF	Reduced cognitive
Respondent 11	Interview	M	WB	63	2005	VF	Memory loss
Respondent 12	Interview	M	WB	58	2017	OM, VF	Memory loss, gait, dysphasia^b^
Respondent 13	Interview	F	WB	52	2010	OM, VF, VP	Memory loss, dysphasia

Abbreviations: FG, focus group; M/F, male/female; OM, ocular motility; VA, visual acuity; VF, visual field; VP, visual perception; WB, White British.

^a^ Respondent 3 participated in a focus group with translational support from friend (Respondent1).

^b^ Respondent 12 participated in an individual interview with translational support from spouse.

The second consisted of four participants, all male, although one participant (9) had to leave unexpectedly before the dialogue commenced. Further participants were unable to attend the two focus groups due to travel and health problems. Table 2 shows the coding tree created following thematic analysis of the transcripts. With few exceptions, the overarching experience of stroke and visual impairment that emerged in respondent accounts was constructed, perhaps unsurprisingly, in terms of “loss.”

**Table 2:** Coding tree centering on the theme of “loss”

Main theme	Subthemes
The physical being	Loss of mobility The multiplicity of problems and healthcare needs
The psychosocial being and “loss”	Self‐identity and spoiled identity Embarrassment, fear, and loss of confidence The financial impact of stroke and loss Loss of agency
The systematic organization of health care	Information giving and lack of vision care Apathy and inequities in practitioner/patient power relations

Embedded within this set of master narratives are a series of subtle, or nuanced, dynamics that constructed a collective understanding of the social world inhabited by this group of individuals, one where personal stories could be located within the body politic of healthcare services and health inequalities. This research presents a critical analysis of research findings through the themes of (a) the physical being, (b) the psychosocial being, and (c) the systematic organization of health care. The core theme illustrates how the lifeworld of the stroke survivors is mediated by concepts such as place, space, and time, as exemplified in contemporary scholarship on the “sociology of the body” (Adelman & Ruggi, 2016). As shown through the stroke survivors’ accounts, connections are made between the experience of stroke, the visual impairments, and health inequalities, by spatial and temporal conceptions.

### 3.1 The physical being

#### 3.1.1 Loss of mobility

Difficulty mobilizing independently after stroke was a recurrent theme from the interviews. The use of a wheelchair following stroke was new to many of the respondents; however, their fears and apprehensions of mobilizing in the wheelchair were heightened when coupled with the newly acquired visual impairments. A notion that mobility issues are worsened for stroke survivors using wheelchairs due to newly acquired visual impairments was furthered through Respondent 11’s dialogue. The stroke causes new and complex mobility issues, but those stroke survivors with subsequent stroke‐related visual disorders are met with additional mobility impairments due to the loss in vision.

They tried me on a big wheeled one [wheelchair] but I could only go round in circles because I could only use one eye. I had to get one… with small wheels and I just drag myself along with my good leg… (Respondent 11).

Language used by the stroke survivors to discuss the loss of a driving license expressed their efforts to retain the ability to drive, like a battle that must be won. The respondents used words such as “surrender,” implying a sense of “giving up,” or defeat from the stroke. Furthermore, this account describes poststroke driving restrictions as culpable to more than one stroke pathology. In this single account, physical impairments from the stroke, poststroke epilepsy, and fear, all contributed to Respondent 8’s loss of driving, thus indicating the multiplicity of barriers that this group perceive they must strive to overcome to continue leading their lives as they did prestroke.

I had driving license… I drove quite a lot and… but my stroke was so bad I couldn’t drive [pause] I had lots of things with my leg and arm and I couldn’t drive [pause] when I got… about two years after my stroke I got [long pause]. I had one epileptic fit… once you’ve had an epileptic fit you can’t drive for another 12 months [pause] then I had two more epileptic fits in the New Year… and then would you know, I had another two epileptic fits in June [pause] the thing is, after that I don’t know whether my legs will be strong enough or if my arms will be strong enough [pause] I might have to surrender it (driving license) (Respondent 8).

Respondent 11 stated that he had driven prior to the stroke, but no longer felt able. This was due to a combination of visual problems and hand–eye coordination, framed more largely by a sense of fear. Furthermore, when discussing the use of buses as an alternative means of getting around, Respondent 11 appeared embarrassed that a bus driver had once asked passengers to disembark to make room for him in his wheelchair, which resulted in a loss of confidence using buses. The below quote indicates how the respondent, in a social context, risks becoming a public spectacle (Foucault, 1975; Wüschner, 2017). Social interaction, and how society accepts or does not accept their newly impaired bodies, mediated many of the discussions around health inequalities. Many of the respondents reported a sense of isolation after stroke, as a result of fear and self‐doubt, which concurs with current research evidence that suggests a loss of confidence with using public transport in relation to vision loss (Gallagher, Hart, O'Brien, Stevenson, & Jackson, 2011).

I did [lose confidence] ’cause when I walked on one bus I couldn’t get in it where he… where he… he wanted me to go in. I couldn’t get in it… and that knocked me a little bit, I thought… is every bus going to be the same (?) I haven’t been on a bus as much since then (Respondent 11).

#### 3.1.2 The multiplicity of healthcare problems

Issues with memory loss were a recurrent conversation in many of the interviews. What is more, the impact of stroke disabilities is so far‐reaching that the respondents can no longer enjoy past hobbies. Respondent 11’s hearing is not impaired, but the multiplicity of health problems prevents him from listening to music in other ways (memory/physical impact on using electronic devices).

… I forget so much [pause] my short‐term memory is terrible… I am terrible with the computer… I’ll do something and then… well an hour or two later I’ll think how did I do it [pause] I’ll spend ages trying to learn how to do it again. I used to mess about with a lot of music at one time… (Respondent 11).

When asked about health inequalities in relation to the experience of having had a stroke, respondent accounts illustrated a range of issues that coincided with the complexity of the relationships that constructed their lives. For Respondent 13, it was important to acknowledge that disturbances in sight and vision, though traumatic, did not have a significant impact on their life immediately after being admitted to hospital following stroke. This was largely due to the fact that during their admission to hospital, they were placed on a hyperacute ward. In these settings, individuals were not ambulatory and relied heavily on members of the multidisciplinary care team.

It was not until the chronic stage of stroke, 6 months from the date of stroke onset, that the visual disturbances became problematic for Respondent 13. National stroke guidelines recommend patients receive a specialist visual assessment during the acute stage of stroke (Intercollegiate Stroke Working Party, 2016), although (Hepworth & Rowe, 2019) this is not available in all acute stroke units. It is possible that patients would not receive the full range of visual therapies unless complaining of visual symptoms at this stage. Respondent 13 described the importance of revisiting visual consequences in the extract below, referring to the everyday activities as “other things.”

But actually 6 months down the line it might be something that’s having the biggest impact on their lives so it’s about… it matters to me now [pause] it didn’t matter to me when there were so many other things that were presenting… but 6 months down the line it matters to me hugely now and its having a massive impact on my life now (Respondent 13).

### 3.2 The psychosocial being

#### 3.2.1 Self‐identity and spoiled identity

One interesting feature of a number of respondent accounts was the deployment of humor in a nonhumorous setting, which seemed to act as a mask for the sense of a damaged self and identity. In the extract below, humor featured prominently to construct how “good care” is described in terms of the shock and distress of having had a stroke. The concept of time, featured as an organizing aspect of talk, centered on special times or seasons that connoted ideas of family, celebration, and happiness. These were powerfully juxtaposed with the descriptor of being a “long‐term patient,” signaling a permanent change in status and identity.

If someone had shot me [laughs]… no I was given good care [pause] as I say I was transferred from stroke to the neuro and it was just that I was there over Christmas and New Year [pause] I was like a long term patient [pause] I had good care… like it felt like very good care (Respondent 6).

For the respondents, a strong feature of the accounts centered on the sense of sexual identity. The quotes below from Respondent 11 support the notion that the male respondents struggled with masculinity and disclosing difficult and intimate aspects of their life without the use of humor. Respondent 11 illustrated struggles with identify after stroke, as he distanced himself from the female staff on one stroke ward and formed relationships with the male nursing staff on another stroke ward. This inference to sexual identify is presented by the respondent's use of masculine language when describing the two contrasting hobbies (rock music vs. a health spa).

…it’s nice because the ward that I used to be on… there used to be a load of male nurses that used to be into rock music so we used to talk about rock music. The first hospital where I had my stroke over in [area name], it was all the young girls, and all they could say was they had a good weekend…[pause] they had gone to a health spa [pause] and I thought… hang on, I can’t imagine that being a good weekend (Respondent 11).

Furthermore, the loss of the stereotypical “male role” from losing employment furthered notions of anger, shame, and loss of sexual identity.

I am on benefits now… I am one of these people that you see on channel 5… they’re always doing… sort of [television programmes]… about people on benefits [pause] I thought… you can’t live my life (Respondent 6).

Employment was further described as more than a job, but a central hub of friendships and networks that fueled their social lives. Following loss of employment, these social networks and relationships were lost also, resulting in social isolation. One's loss of employment can, therefore, have a significant impact on other aspects of the respondent's self‐identity. The wider social aspect of work was further lost after stroke for many of the respondents, in the way that sociologists have described “social death.” This loss of employment therefore links to social isolation and a sense of invisibility. The account below describes the knock‐on effect of losing employment, to affect social networks.

… it changes your life altogether because when you’re working… it’s not just a case of working [pause] the people you associate with… well you think they’re your friends pause] you think… this stroke is catching… you don’t see them again (Respondent 11).

#### 3.2.2 Embarrassment, fear, and loss of confidence

A recurrent theme that emerged from the transcripts was the fear and embarrassment that the stroke survivors experienced from new visual impairments, including the hallucinations of Charles Bonnet syndrome described by Respondent 13. This fear resulted in further isolation, as she “lived in fear” of being “found out” and did not want to risk being told that her mental state had been compromised after stroke. The act of concealing these symptoms from family and medical staff signifies isolation and fear while attempting to cope with new impairments.

…things just appeared in front of me. I thought I was going mad and I wouldn’t tell anybody about it [pause] I thought they’d lock me up. Why the hell would you tell somebody? At first I thought they [hallucinations] were real and then I realised from people’s responses that they weren’t real [pause] and once I realised that I actually thought I’d gone mad so I kept very, very quiet about them (Respondent 13).

This theme of fear and embarrassment brought specific insight into the lives of the respondents after stroke, and how they adapted to, or even masked, their impairments to reclaim dignity and acceptance. The respondents understood the benefits of a white cane in warning the public of their visual impairments, described below as a “parting of the waves” when used in public spaces.

A white cane just gets you… it’s like the parting of the waves… people just move out of your way all the time [pause] people are so courteous to you when you have got that white stick in your hand but they are not as courteous when you’ve got a walking stick [pause] like if you’ve got a white stick and you get on a bus people will get up and give you their seat but if you walk on with a walking stick they won’t [pause] it’s a hell of a difference (Respondent 6).

The respondents stated that their visual impairments were “hidden” from society, making it difficult to gain public support. Therefore, a white cane would appear an obvious support to most; however, it was later revealed that many refused to use one. This practice may be illuminated through sociologist Erving Goffman's theory of “passing” spoiled identities (Goffman, 1968), which states that one can manage their “stigma” by concealing the visible indications of the impairment. It is therefore possible that respondents perceive the cane to be a public marker of their visual impairment and thus reject the cane in a bid to retain their prestroke social standing.

…so there is something to say about not taking help either… I was offered sticks to walk with…but I wouldn’t accept. (Respondent 13)

#### 3.2.3 The financial impact of stroke and loss

The personal cost of stroke to the respondents was a prevalent topic of discussion. This topic emerged from discussions in relation to the cost of losing employment, and the psychosocial impact of loss of income and self‐identity. The loss of employment, and subsequent income, coupled with greater expenditures after suffering stroke, presented as an escalation of inequalities. Respondent 7 recounted the link between loss and income and greater expenditure. Namely, the greater expenditures after stroke took the form of public transport costs required to travel to the multitude of hospital appointments.

Well, you’ve got less income and more expenditures, it’s not very good is it? (Respondent 7)

It was apparent that many were still feeling the financial burden from loss of employment. This burdensome feeling was described by Respondent 13, as she could no longer work, and her husband left work to care for her. This manifested later in the form of depressive symptoms, as she described it (the financial loss) all “became too much.”

My husband actually finished work to care for me… we didn’t have my job but we didn’t have [husband’s] job either…we thought we were going to lose the house originally… I had an absolute crash, I just decided I didn’t want to live anymore. (Respondent 13)

The lengthy extract below from Respondent 11 portrays an attempt to justify his current social and economic position, by recounting his previous income in depth, before describing a complete loss of income. This complements Goffman's theory of “impression management” (Goffman, 2004), whereby one employs techniques to stage the character that they wish to perceive to others. It can be inferred, then, that Respondent 11 was, to some extent, ashamed of his current financial position and attempted to influence external perceptions of his life. The final statement of this account is a powerful ending to the narrative: “I lost all that,” indicating how quickly his life changed financially after the stroke.

…before I had my stroke I just went from one company to another from £280 a week [pause] and… one of the bosses had moved to this other company and he asked me to go with him…and he said for the same job we’ll give you £350 a week [pause]and I said yeah I’ll take that (laughs) and then within I think it was 2 months I got a day job with another company and the owner of the company said… I’ll give you £400 a week [pause] I thought yeah ok… I thought £400 a week was decent (laughs) but yeah… I lost all that (Respondent 11).

Respondent 10 described his journey from being self‐employed, to a reliance on government benefits. Therefore, this issue of a reduced income was further explored in terms of a change in social status and self‐identity. That is, the way that work is an important part of how people develop a sense of worth and social value. Labor is productive, and earning money defines one's spending power as a consumer. This could be linked to the centrality of work in Marxist social theory (Marx, 2010), which states that human society progresses through a struggle between two distinct social classes.

I was self‐employed, well in a way, because I had to go straight on… what’s it called… DSA [Disability Support Allowance]? (Respondent 10).

#### 3.2.4 Loss of agency

When describing the stroke condition, the concept of stroke care was expressed in terms of being “good” or not. This concept emerged as an issue worthy of further exploration, an avenue of inquiry that identified, or problematized, the criteria against which service user decisions were mediated by clinical–medical power relations, statuses, and the dominance of expert knowledge. The centrality of the statement, “I’m not a doctor,” in the data extract below, makes explicit the deferential disjuncture between medical and lay language.

No… I just don’t know because you know… I’m not a doctor… I don’t know [pause] even if someone had told me I’m not sure I would have taken it in or been in a state to understand it [pause] yeah so it’s very… very difficult because what can you do if I’m… basically it would make no difference or maybe I would have understood some of it but I just don’t know (Respondent 7)

Very much like Parson's concept of the “sick role,” whereby, in order to be “sick” one must enter a role of sanctioned deviance (Segall, 1976), Respondent 10 appeared to adopt and play a dramaturgical part on the stage of the clinical/medical theater. Interaction was contextually framed in terms of compliance with medical discourse. To “nod in the right places” does not signal an equitable relationship, rather one premised on medical power and prestige. It appears that the respondent was talking about doing things that were expected of him at that point in time. The gesture of concurrence (nodding) can be seen as a ritualized response rather than being indicative of any real understanding of what was being said.

…No I just accepted it straightaway… when they done the tests, you know, I just knew, even now, it’s like looking through a cloud [pause] what they (doctors) were saying… I was nodding in the right places… but it… wasn’t staying in there [pause] I wasn’t grasping what they were saying sort of thing… it was going in and just going round (Respondent 10).

### 3.3 The systematic organization of health care

#### 3.3.1 Information giving and lack of vision care

The key inequalities identified from the respondent accounts related to a lack of information, the respondent's inability to understand information in the format it was provided, and the subsequent reliance on family members to retain information, which furthered previous suggestions of a loss of independence. Variation in information provision mainly related to the variation in hospital staff's specific knowledge and experience of stroke‐related eye conditions. Hospitals with reportedly noneye trained staff provided little or no information to the stroke survivors. Respondent 2 offered a terse response when asked about written information or advice provided, initially, in the hospital regarding visual impairments. She reported a lack of integrated stroke, and visual care was to blame for the lack of information.

In a word… no [pause]I got very little information [pause]… some hospitals didn’t have anything to do with any stroke or anything and the last thing would be eye problems and then other hospitals, like [hospital 3], are really good. (Respondent 2).

The respondents contested the format and timing through which information concerning their visual impairments was delivered to them. Information given verbally (“face‐to‐face”) was deemed preferable by some, but generated concern for others in terms of memory retention. Although written information was not always useful for the respondents, they reported that this could be passed to family. Therefore, timing and format of information appeared highly dependent on the individual's stroke impairments.

I would have liked a letter because I can give it to someone and they can read it out to me… because if I got a phone call, they [family] would say, ‘what did they say?’ and I’d say, ‘I don’t know’ because I’ve forgot. (Respondent 9).

When asked about hospital appointments for visual and general stroke rehabilitation offered after the discharge from the stroke ward, respondents reported contrasting experiences: Either they received no poststroke care or were inundated with a multiplicity of appointments. While Respondent 10 was offered no poststroke visual rehabilitation, he spoke of attending multiple appointments for his stroke condition, which subsequently affected his life financially, due to the incurred travel expenses in attending the hospital. Moreover, the extract below describes his reliance on his care home to take control of travel and appointments. Respondent 10’s account can be related to changes in spatial (space) and temporal (time) domains as a result of having had a stroke. He portrays a world without clear temporal markers: an unending repetition and routine that characterizes life in/at the “home.”

… no… I would have forgot a lot of them [appointments]. It’s like… my tablets… I’ve got to have a nurse that gives me my tablets ’cause I forget them [pause] I know what tablets I’m getting when you give me them but I… I forget them ’cause I… every day seems to run into, into one, you know… so (Respondent 10).

#### 3.3.2 Apathy and practitioner/patient power relations

The stroke survivors talk about their stroke disabilities with a sense of finality and accepted it “straightaway.” This language resonates a sense of surrendering, of giving up without a struggle. On several occasions, respondents used the term “it comes with the territory” when discussing their stroke impairments, suggesting a common sense of acceptance of defeat across the stroke community.

Well I just accepted it straightaway… I thought it had just come with the territory of the stroke (Respondent 1).

Respondent 8 (below) is describing his physical therapy after stroke. He provides an emotive account of powerlessness, trapped in his own body, “I couldn't speak… I couldn't use my arm…”. He compares different therapies by the length of time they took, again showing the respondent's use of spatial and temporal language to describe their life after stroke. His description, “I thought, I have got to get them out,” implies frustration from loss of agency and his determination for independence. A strong priority for this respondent, therefore, was patient‐centered stroke care, whereby he chose goals that were important to him at that moment in time (making dinner and walking short distances independently), which he then interpreted as achievements that gave him hope and motivation. This approach to stroke care, which gave the patient control of their rehabilitation, ultimately reduces the perceived patient–clinician power imbalance.

…for my leg [pause] really, I had nothing… I couldn’t speak… I couldn’t use my arm and I couldn’t… I used to have someone come in to make my dinner and I thought I have got to get them out… I will make my dinner… but I was thinking I can’t bloody make my dinner because I can’t butter… I can’t you know, but I can make it… it’s given me hope that I would go this further and walking down the hallway… and then walking down the hallway twice… and then walking down the hallway and into the kitchen in the house… and then when I went into the street and I got further and further [pause] I had just put my hand in my pocket and that was it… I wasn’t going to speak to anyone and I just make my leg better (sic) (Respondent 8).

Furthermore, this respondent's description (above) of “I had just put my hand in my pocket and that was it…” refers to his paretic arm that he put in his pocket (out of public sight), therefore concealing the disability. Additionally, stating, “I wasn't going to speak to anyone” while walking down the street, illustrates that his speech and arm impairments were still problematic; however, while he was concentrating on improving his walking ability, the other impairments were put to one side. This account reflects the patient‐centered care approach, shown through the respondent's determination to concentrate on one task at a time, driven by his own personal priorities. What is more, Respondent 8’s actions in concealing his stroke impairments could be depicted as further examples of passing a spoiled identity.

## 4 LIMITATIONS

Focus groups and individual interviews may yield differing results, and so combining these may present a study limitation. However, focus groups were not always feasible within the visually impaired stroke cohort, and a flexible approach was necessary, offering individual interviews as an alternative to support the needs of the stroke survivors and still capture their experiences. The same interview plan was used for focus groups and interviews, following a topic guide, to reduce potential bias.

Furthermore, the gender differences in the two focus groups could have impacted on the findings. The female participants in the first group naturally chose to attend with their acquaintances, and discussion was therefore easier for them. The male participants were not as familiar, and conversation was initially quite stifled. As group dynamics may affect the success of focus groups, it may have been more beneficial to mix the groups to produce richer data. Future research should consider careful selection of individuals for each focus group to aid control of the group dynamic. In addition, the ethnic diversity within the sample was a limitation of this research, and future studies should aim to employ purposive sampling methods to target the wider ethnic communities.

## 5 DISCUSSION

The findings from this research explored the longer‐term, lived experiences of stroke survivors with visual impairments, to investigate the impact of living with any inequalities. Overall, the experiences of the visually impaired stroke survivors comprised of inequalities, which centered on the overarching theme of “loss.”

Inequalities concerning the physical being described the loss of virtual possessions after stroke, in addition to the loss of the respondents’ ability to mobilize and undertake everyday tasks as freely as they could prior to suffering stroke. The recounted loss of so many aspects of one's physical life following stroke and/or visual impairment concurs with earlier research that highlighted cases of transport difficulties and loss of employment due to stroke/visual impairment (Cumberland et al., 2016; Gallagher et al., 2011). Furthermore, accounts of “loss” in terms of meaningful activities, due to visual impairments within nonstroke cohorts, have been reported previously in qualitative studies (Stevelink, Malcolm, & Fear, 2015; Teitelman & Copolillo, 2005), highlighting the predominance and significance of this occurrence.

However, in the current research the respondent accounts expressed strong links between the various physical losses, such as loss of driving influencing loss of employment, and the psychosocial losses of identify and confidence due to loss of earnings. Although it has been widely stated that socioeconomic disadvantage precedes poor health (Wilkinson, 2002), links to “reverse causation” have been noted at “an individual level,” whereby poor health and social problems affect loss of earnings (Lynch et al., 2004). This phenomenon has been described as a “cascade of health disparities” in previous research concerning adults with learning disabilities (Krahn & Fox, 2014; Krahn, Hammond, & Turner, 2006), but seems equally pertinent to inequalities suffered following stroke. Therefore, to reduce inequalities, health disparities at all levels must be considered and addressed. If clinicians are made thoroughly aware of the possible impact that stroke and visual impairment can have on patients, at all stages of their lives, including those outside of the clinic room, then they can prepare patients and signpost to support services that could reduce travel and financial complications.

The second theme that emerged described inequalities concerning the psychosocial being, namely a loss of self‐identity and loss of agency. Goffman (1968) described the concept of a “spoiled identity” as living with a discrediting attribute, a concept originally published by Mead (1934). The psychological effect of stroke and the consequential loss of one's identity have been described previously. Themes of loss and identify have been noted in qualitative research, combining focus groups and interviews with stroke survivors, due to loss of engagement with previous activities, which contributed to their personal identity (Clarke, 2003). Furthermore, Murray and Harrison (2004) reported themes of disrupted embodiment and loss of self when interviewing stroke survivors. These views are comparable to those of the current study participants, who struggled to come to terms with their new poststroke bodies and the outward markers that display their disabilities. The respondent accounts discussed in this research conveyed emotional consequences of coping with a new, spoiled identity.

Little is known regarding spoiled identity following visual loss, which for some of the respondents was their only remaining physical impairment in the years following their stroke. The respondents described the “invisibility” of visual impairments through apprehensive and fearful accounts. The respondents noticed differences in the public's acceptance and curtesy of outwardly visible impairments. However, such acceptance was not always offered to those living with solely visual impairments that presented no outward marker of disability. For example, Respondent 11 described a situation where passengers were asked to disembark a bus to allow him on with his wheelchair (his visible marker of impairment).

The respondent accounts, therefore, suggested that living with a visual impairment “protects” against a spoiled identity, although for this group, a visible marker of impairment would, potentially, be of greater benefit in gaining public support and assistance. However, when later discussing the use of a white cane, which is a purely visible indicator of impairment, the respondents collectively refused. Their accounts were consistent with the theory of “passing” a spoiled identity through impression management (Goffman, 1968, 2004; Millen & Walker, 2001), by refusing to accept a supportive aid that could be observed as a marker of a spoiled identity. The act of “impression management” assumes the use of coping strategies for “passing” spoiled identities, described as social interactions adopted by the affected persons, to “normalize” themselves in the presence of “normal others” (Goffman, 1968).

This concept of impression management can be detected in earlier qualitative work by Teitelman and Copolillo (2005) with visually impaired groups, from the proactive adaptations made to cope with the emotional impact of vision loss. The authors reported that strategies, such as humor and comparing themselves to others worse off, were employed by the participants to restructure their views of vision impairment in a positive light. Additionally, humor was frequently used by the current (male) study participants in nonhumorous settings, suggesting a coping strategy when disclosing intimate, poststroke adversities (Wilkinson, Rees, & Knight, 2007). This concept is further supported by stroke‐specific literature, which identified humor as a useful tool for coping with the difficulties experienced after stroke (Ch'Ng, French, & McLean, 2008; Wilkinson et al., 2007). These findings present the emotional difficulties that stroke and vision impairments produce separately, while our current study has highlighted the combined impact to those suffering from poststroke visual impairments. This analysis exemplifies the severity of their emotional turmoil and furthers previous notions of shame that respondents associate with their lives after stroke. Furthermore, the use of humor due to discomfort caused by a perceived power imbalance may suggest a limitation of this research, despite efforts made to control for power imbalance during the study design.

Additionally, shamed and masculine language was observed in male respondents’ accounts describing loss of agency, implying a loss of the stereotypical male role (their prestroke identity). This idea that gender influences one's self‐identity, and thus is worthy of preserving, is furthered by philosopher Judith Butler's theory on “gender performativity” (Butler, 2002). Butler (2002) argued that one may overplay their gender role when their identity is threatened, which offers a possible explanation for the masculine language observed during the interviews with male respondents when describing their trepidations caused by the stroke.

Comparably, gender has been presented as a possible factor in the uptake of health care, postulating that males tend to demonstrate poor help‐seeking behaviors (Corney, 1990). This was observed where primarily male respondents refused to accept physical aids, such a white cane, and, instead, chose to live with the additional burden. Therefore, it is possible that perceived societal expectations of the male role, noted in this research, could further affect stroke patients by creating a barrier to voluntarily seeking or accepting health care, despite a known need, due to embarrassment (Pinkhasov et al., 2010; Smith, Braunack‐Mayer, & Wittert, 2006).

These findings, therefore, suggest that an inequality exists where patients who have suffered a stroke have to cope with this additional social “trauma” through loss of self‐identity, compared with those that have not. Consequently, this group are at greater risk of “hidden” physical (e.g., visual defects) and “psychosocial” (e.g., depression) impairments (Taule, Strand, Skouen, & Raheim, 2015). Not only do stroke survivors have to adapt to their new disabilities, but they are also burdened with the insistent efforts required to conceal their impairments from the public, and in some accounts, even their families. This subsequently results in unaddressed and unmanaged impairments, which have been shown to impact on the patients’ health and quality of life (Ali et al., 2013; Jones & Shinton, 2006).

Recommendations from similar research studies have suggested offering longer rehabilitation periods to stroke survivors, to allow for time to adjust to their new “selves” with the necessary psychological support networks in place (Ellis‐Hill, Payne, & Ward, 2000; O’Connell et al., 2001). Taule et al. (2015) further identified a need to support stroke patients in making sense of their new, altered bodies, and processing the emotional reactions caused by a changed body, through their interviews with stroke survivors and healthcare professionals. In addition, similar research studies have produced recommendations for clinicians to review the discharge materials given to patients to ensure pertinent support is highlighted at this early stage (O’Connell et al., 2001), which appears applicable to the current study, as participants identified poor information provision after stroke regarding visual rehabilitation and long‐term support. These findings, in combination with the current literature base, highlight a clear desire from both clinicians and visually impaired stroke patients for better support informing them of what they can expect after stroke, and offer advice on how to adjust to their new impairments long‐term.

The final theme identified from the stroke survivor accounts considered inequalities in the organization of health care after stroke, which included issues with stroke and vision services, mainly a lack of care, or health care that did not meet the needs of the stroke survivors’ new impairments. Earlier research has reported a significant health inequality in the delivery of visual care after stroke, which was frequently unavailable or inadequate (Rowe, 2013). This inequality was observed by the stroke survivors who recognized differences through their own experiences in the care offered, dependent on the hospital site.

A strong theme of apathy and acceptance came through in all interviews, which followed on from previous discussions of loss of agency after stroke and vision loss. This apathy, coupled with the respondents’ inabilities to mobilize or communicate effectively, resulted in social isolation and the loss of relationships and social networks. The consequence of social isolation after stroke has been well established in the literature (Haley, Roth, Kissela, Perkins, & Howard, 2011), with means of community integration and peer support groups recommended to counteract feelings of isolation and build resilience (Sadler, Sarre, Tinker, Bhalla, & McKevitt, 2017). One broad suggestion was to “accommodate all existential aspects of stroke recovery” (Taule et al., 2015), which could be interpreted as the use of person‐centered care models in stroke recovery.

However, many of the respondents identified specific problems with accessing care due to their impairments. The respondents identified issues with reading hospital appointment letters, transport difficulties—including cost implications associated with travel—and a lack of home‐based care for visual rehabilitation. Issues with reading written information have been reported in earlier qualitative studies with stroke survivors, where it was identified that vision impairment impedes the person's ability to read hospital information offered to them in written form, resulting in communication breakdown between staff and patients (O'Halloran, Grohn, & Worrall, 2012). To counteract this issue, the authors suggested that the wider stroke care team share experiences and observe each other communicating with stroke patients, to learn how to achieve effective communication despite disabilities. It could be inferred that a similar approach is adopted to counteract the issues described in the current study, whereby healthcare professionals and stroke survivors work together to ensure information is documented in a way that they understand, on an individual‐needs basis.

Furthermore, concerns were exacerbated through the multiplicity of health appointments offered, and the stroke survivor's inability to interpret hospital letters, or commute to appointments. A possible solution to these attendance issues is the implementation of patient‐centered care models.

Patient‐centered care allows the patient to regain ownership of their physical recovery and reduces dissatisfaction that arises from disappointing recovery (Wiles, Ashburn, Payne, & Murphy, 2002). The NHS recommends offering care tailored to the individual's needs; however, in practice this is not always followed, with NHS England (NHS England., 2015) identifying barriers to supporting this care approach to include lack of time, inefficient support services, and inadequate clinician skills. A clear recommendation from this research is for clinicians to discuss the range of visual and psychosocial rehabilitation options available after stroke, offering their recommendations based on clinical experience, but ultimately placing the patient at the center of the decision‐making process.

The findings from this research identified numerous accounts of power imbalance within the healthcare setting. This concurs with O'Halloran et al. (2012), where power imbalance was reported through inefficient communication between stroke patients and their physicians, resulting in feelings of embarrassment. This patient‐centered care approach recommends shifting the power to the patient so that they can assume responsibility for managing their own condition (Cott, 2004), and not be pre‐empted by clinicians.

This research has identified that stroke survivors suffer from a vast range of physical and visual disabilities following stroke, which subsequently results in different preferences in how information is communicated according to the individual's needs and requirements. It would appear to be beneficial to the patients for these needs to be addressed prior to hospital discharge, and so the appropriate form of information and communication can be used. However, it may be of further benefit to revisit the patient's preferred choice of communication during their rehabilitation process, as their needs may alter as their disabilities change over time.

## 6 CONCLUSION

In summary, the collective theme identified from the interviews with visually impaired stroke survivors considered “loss” in many forms. Loss of physical aspects of the respondents’ lives after suffering stroke‐related visual impairments was found to subsequently impact on the loss of their psychosocial being, as many of these physical loses were attributed to their self‐identities.

Furthermore, the findings from this research highlight the longer‐term implications of stroke‐related visual impairments, beyond those collected in the clinic setting, which appear to go unrecognized and unmanaged in many cases. Examples of such implications include the social isolation described by many of the respondents, due to fear and lack of confidence. These results emphasize a need to inform and educate both clinicians and stroke survivors of the bigger picture of life after stroke, indicating what is to be expected and highlighting what support is available to patients following hospital discharge. The wider stroke multidisciplinary team should strive to reduce power imbalances, through effective communication means adapted to the needs of the stroke survivors, and using a patient‐centered care approach in all areas of rehabilitation. An unmet need was noted where not all stroke survivors are offered visual rehabilitation after stroke, and this inequality must be addressed nationally to ensure equal care for all.

## CONFLICT OF INTEREST

The authors have no conflicts of interest to declare.

## AUTHOR CONTRIBUTIONS

One author (KH) recruited the participants and conducted the focus groups and individual interviews. As experience in qualitative interviewing can impact on whether rich data will be obtained, the author (KH) underwent additional training and was, further, closely supported on this work by the other highly experienced authors. Two authors (KH and DM) conducted the analysis, and all authors contributed to the writing of this article.

### Peer Review

The peer review history for this article is available at https://publons.com/publon/10.1002/brb3.1738.

## Data Availability

The data that support the findings of this study are available on request from the corresponding author. The data are not publicly available due to privacy or ethical restrictions.
